# Lineage Tracking for Probing Heritable Phenotypes at Single-Cell Resolution

**DOI:** 10.1371/journal.pone.0152395

**Published:** 2016-04-14

**Authors:** Denis Cottinet, Florence Condamine, Nicolas Bremond, Andrew D. Griffiths, Paul B. Rainey, J. Arjan G. M. de Visser, Jean Baudry, Jérôme Bibette

**Affiliations:** 1 Chemistry Biology Innovation (CNRS UMR 8231), École supérieure de physique et de chimie industrielles de la Ville de Paris (ESPCI ParisTech), PSL* Research University, Paris, France; 2 New Zealand Institute for Advanced Study, Massey University, Auckland, New Zealand; 3 Max Planck Institute for Evolutionary Biology, Plön, Germany; 4 Laboratory of Genetics, Wageningen University, Wageningen, The Netherlands; Fred Hutchinson Cancer Research Center, UNITED STATES

## Abstract

Determining the phenotype and genotype of single cells is central to understand microbial evolution. DNA sequencing technologies allow the detection of mutants at high resolution, but similar approaches for phenotypic analyses are still lacking. We show that a drop-based millifluidic system enables the detection of heritable phenotypic changes in evolving bacterial populations. At time intervals, cells were sampled and individually compartmentalized in 100 nL drops. Growth through 15 generations was monitored using a fluorescent protein reporter. Amplification of heritable changes–via growth–over multiple generations yields phenotypically distinct clusters reflecting variation relevant for evolution. To demonstrate the utility of this approach, we follow the evolution of *Escherichia coli* populations during 30 days of starvation. Phenotypic diversity was observed to rapidly increase upon starvation with the emergence of heritable phenotypes. Mutations corresponding to each phenotypic class were identified by DNA sequencing. This scalable lineage-tracking technology opens the door to large-scale phenotyping methods with special utility for microbiology and microbial population biology.

## Introduction

Populations of microbes are increasingly used as experimental tools to study evolutionary processes [1, 2]. To monitor evolutionary change competition experiments between neutrally-marked evolved strains and ancestral strains are often used [2, 3]. These assays provide a measure of the fitness improvement of derived clones or populations, but do not reveal phenotypic or genetic changes. In recent years, technological advances in DNA sequencing have made it possible to track evolution at the level of individual mutations [4–6]. However, connecting mutations to their phenotypic effects remains a significant challenge, often requiring years of painstaking genetic and physiological analyses [7, 8]. Desirable are methodologies that allow detection of subtle phenotypic differences at the same level of resolution as provided by deep sequencing. To date, a handful of studies have exploited fortuitous differences in colony morphology among variant types and adaptive phenotypes after plating samples on agar media [9–11]. Detection of mutant types based on differences in colony morphology requires that adaptive mutants affect morphology and reach sufficient frequency to be detectable by plate culture. If it were possible to track heritable phenotypes in the descendants of many individual cells, then subtle heritable phenotypic differences among cells could be discerned within an evolving population. Ideally, these different cells would be non-destructively sorted and coupled to DNA sequencing to detect and to understand genetic and phenotypic evolution.

**Fig 1:**
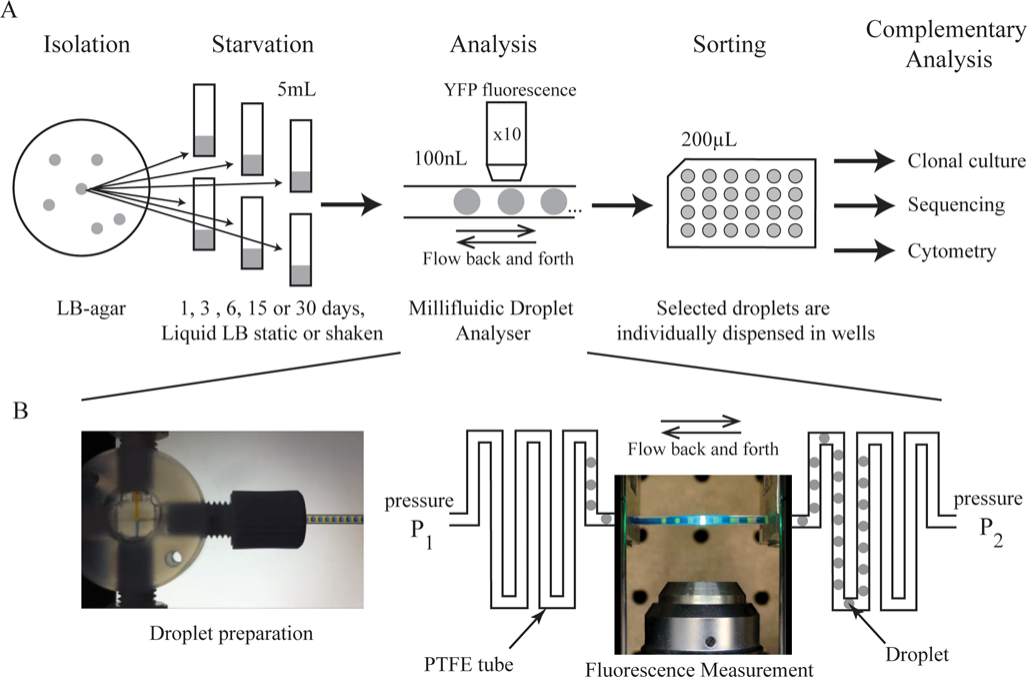
Schematic of the study. (A) Six *E*. *coli* clones were isolated from growth on LB agar. Each was used to inoculate six sealed tubes of 5 mL liquid LB. The resulting populations were maintained in starvation for 0, 1, 3, 6, 15 or 30 days. Populations were then analysed by growing colonies from single cells in drops of 100 nL fresh LB medium. Individual drops were collected in microplate wells for further analysis with complementary methods. (B) Millifluidic droplet analyser principles. Droplets are prepared in a cross junction (see S1 Movie) and stored in a PTFE tube (500μm inner diameter). By controlling the pressure at each side the sequence of drops can be moved back and forth in front of the measurement point (see S2 Movie).

Here, we use a drop-based millifluidic system (Fig 1) that allows the compartmentalization of individual cells in ~100 nL drops, which function as independent microreactors [12–14]. While the growth of cells in emulsion droplets is not new [15, 16] the present system allows the descendants of hundreds of independently isolated cells to be tracked for ~15 generations. This allows heritable phenotypic changes in individual cells that are undetectable at the single cell level to be exponentially amplified and quantified. To demonstrate the utility of the technology, we monitored the evolution of a single *E*. *coli* clone carrying a yellow fluorescent protein (YFP) reporter [17, 18] during 30 days of starvation. During prolonged stationary phase, populations are expected to evolve and diversify. Indeed, previous analysis of such populations was instrumental in the discovery of Growth Advantage in Stationary Phase (GASP) mutants [19–22]. Moreover, in the absence of shaking, evolving populations may become heterogeneous due to alterations in the selective environment wrought by metabolic changes in the founding population and spatial heterogeneity [9, 23]. In general, depletion of the nutrients by one dominant clone may open opportunities for new mutants to arise that take advantage of changes in ecological opportunity [22, 24].

## Results

We demonstrate the utility of the millifluidic colony analyser to monitor heritable phenotypic changes at high resolution by studying changes in populations of *E*. *coli* propagated under conditions of starvation over the course of 30 days. The experiment involved growth of six replicate populations under static culture conditions, and three replicate populations under shaken conditions, for a total of 30 days. At set intervals (days 0, 1, 3, 6, 15 and 30) independent microcosms were destructively harvested and cells were analysed in order to detect heritable phenotypic diversity (Fig 1A).

The millifluidic colony analyser [12,13] is based on droplet-based microfluidics [14, 25, 26]. A detailed description of the technology is available in previous publications [12,13]. Two distinct phases are embedded in a continuous flow of fluorinated oil in millimetre-scale tubing (inner diameter of 500 μm). Each nutrient drop reservoir (~100 nL) is separated within the “train” from its neighbour by a hydrocarbon drop of the same size. The train of drops flows continuously (Fig 1B, S1 and S2 Movies) ensuring that the contents of each drop are continually mixed. This also ensures that a thin layer of fluorinated oil always lubricates the interface between the drops and the surface of the tube. Before injection into the device, bacteria are diluted with fresh medium so that, assuming a Poisson distribution of cells in drops [27, 28], the average number of cells per drop, *λ*, is ~0.5 and most droplets contain either no cells, or just a single cell. For a given sample, the founding inoculum size is readily deduced from the fraction of droplets showing growth (see Materials and Methods). About 1,000 droplets were maintained in a single tube, 10 meters long, and approximately one droplet in two displayed growth (~500 inoculated droplets per experiment). Lineage tracking is based on fluorescence measurement. The *E*. *coli* clone used to initiate the evolution experiment carries a chromosomally-integrated yellow fluorescent protein (YFP) [17, 18]. YFP fluorescence is measured in each drop every 10 minutes (S2 Movie). For each single cell the fluorescence signal is measured during the course of growth. Expression of YFP is driven from a constitutive *lac* promoter (see Materials and Methods) [17, 18], but is nonetheless responsive to changes in the metabolic status of the cell, for example, those wrought by mutation. This provides a sensitive read-out of phenotype [29]. By tracking the descendants of individual cells, minute heritable changes in growth, yield, and YFP expression stand to be amplified, thus allowing discrimination between phenotypes that are otherwise indistinguishable at the single cell level.

**Fig 2:**
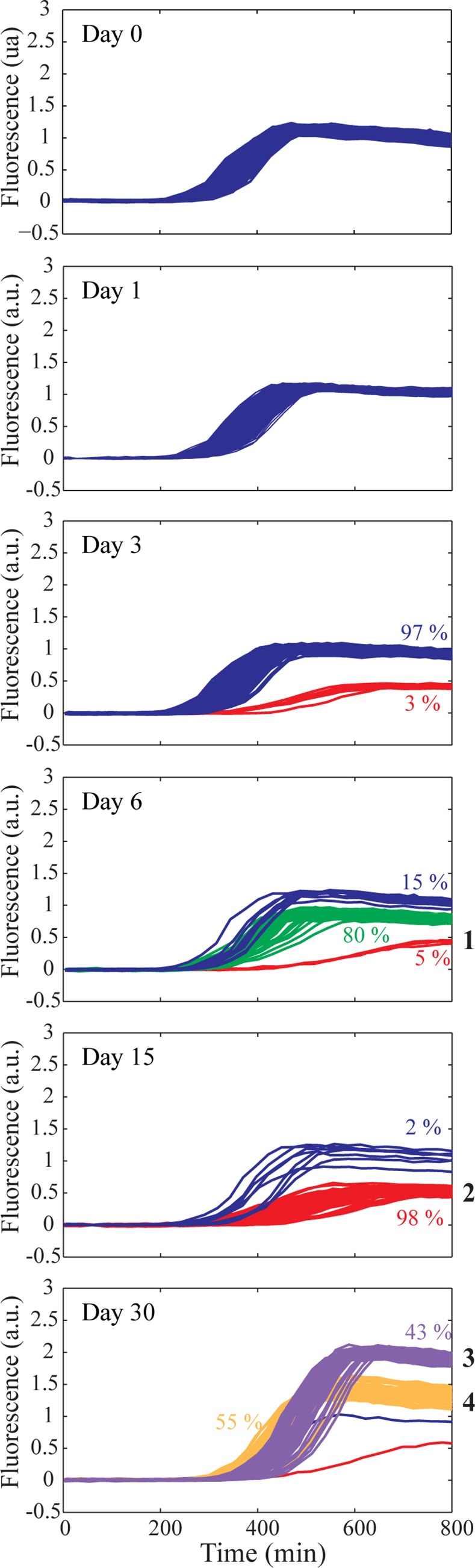
YFP fluorescence profiles obtained by lineage tracking. Samples of ~500 cells from independent evolving populations from a single replicate experiment were obtained for the ancestral population (day 0) and after 1, 3, 6, 15 and 30 days of starvation in static microcosms (from top to bottom) and grown for ∼13 h in individual droplets. A.U. is arbitrary units. Measurement of YFP fluorescence from each droplet was measured every 10 min. Phenotypic classes sampled are labelled 1–4 (see text). Colors discriminate phenotypic classes. See S2 Fig for profiles from five additional replicate starvation experiments.

At successive time intervals, a sample of cells from static and shaken microcosms was collected and analysed with the millifluidic machine (Fig 2; S1 and S2 Figs). Analysis of ~500 occupied drops inoculated with the ancestral *E*. *coli* strain (on entry to stationary phase) showed that the increase in fluorescence acquired over 15 generations of growth (from 1 to 10^5^ bacteria), is identical for all drops (Fig 2). Indeed, the coefficient of variation (CV) in the millifuidic system (5%) is greatly reduced compared to the CV using flow cytometry (34%) (S3 Fig).

In the first selection experiment based on populations maintained in static broth microcosms (Fig 2), after 1 day, a single phenotype was observed. Fluorescence profiles were homogeneous. At day 3, two distinct profiles were detected: one with the same final fluorescence intensity as the ancestor (blue lines), and a new type with a lower final fluorescence intensity (red lines). At day 6, three profiles were detected: two with the same fluorescence intensity as observed at day 3 (blue and red lines), with one displaying an intermediate intensity (green lines). At day 15, two main classes remained evident (blue and red lines) with the green type no longer detected, however the derived population (red lines) represents 98% of the population. At day 30, two new profiles were detected (purple and yellow lines) with a single droplet harbouring populations manifesting phenotypes of the two previously dominant classes from day 15 (blue and red lines).

Next we asked whether the different fluorescence profiles have a mutational cause. The millifluidic device allows the recovery of individual droplets associated to different fluorescence profiles (S4 Fig). Individual drops corresponding to four different classes (numbered 1 to 4 in Fig 2) from day 6 to 30, were plated on LB-agar. They produced distinct colonies with varied morphology (S5 Fig) that were distinctly different to that of the ancestral strain. Types isolated at day 6 and day 15 of static starvation (phenotypes 1 and 2) had a mucoid colony morphology [30], whereas colonies isolated at day 30, corresponding to phenotypes 3 and 4, showed ancestral-like morphology, but the diameter of each colony was respectively smaller, and larger, than that of the ancestor. To test whether these changes were heritable, cells from droplets expressing altered phenotypes were propagated in LB broth and the phenotype rechecked both in drops and via agar-plate culture. In every case the phenotypes mirrored those of the parental types.

In order to determine the genetic basis of the different profile types, we performed whole genome sequencing on four clones from each of the four phenotypically distinct derived populations (Table 1). Mutations were found within clones representative of each profile. The four clones of phenotype 1 with a mucoid colony had identical genotypes, containing just a single point mutation in *yrfF* (G99V). Little is known about *yrfF* but deletion is known to induce mucoidy [31]. All four clones of phenotype 2, which also produce a mucoid colony on agar plates, also contain a mutation in *yrfF* (Y98D), however the position of this mutation is different from that in phenotype 1. The difference reflects the fact that each test tube is an independent biological replicate, but the gene-level parallelism points to evidence of selection and thus an adaptive effect of these mutations [8, 32]. In addition, all replicates of phenotype 2 carry a mutation in *wzc* (D642G), a further known contributor to the mucoid phenotype [33]. For phenotype 3, all four genotypes contain an insertion of IS5 between *csrA* (regulator of glycogen synthesis and catabolism) and *alaS* (alanine synthesis). IS5 movement is known from prolonged stationary phase experiments [34]. In contrast to the first three phenotypes, where all the clones shared mutations, for phenotype 4, one genotype had a unique mutation in *barA* (D472N) and the other three clones each had a different mutation in *mglC*. No mutations were observed in *lrp* or *rpoS*, which are characteristic of Growth Advantage in Stationary Phase (GASP) mutants [35]. Nevertheless, the mutations in *barA* and *csrA* are related to global regulation and are reminiscent of *rpoS* expression modifications. *barA* has been reported to regulate *rpoS* expression [36], while *crsA* play a role in the regulation of *barA* [37, 38] and represses genes expressed in stationary phase [39].

**Table 1:** List of mutations detected in sequenced clones that define phenotypes 1, 2, 3 and 4 (see Fig 2).

	Clone	Mutations					
	1	YrfF G99V					
Phenotype 1	2	YrfF G99V					
(Mucoid, Day 6)	3	YrfF G99V					
	4	YrfF G99V					
	1	YrfF Y98D	Wzc D642G	MenB M56I	IS2 *ompT/pauD*	IS5 *htrL/rfaD*	YcgE E71V
Phenotype 2	2	YrfF Y98D	Wzc D642G		IS2 *ompT/pauD*	IS5 *htrL/rfaD*	
(Mucoid, Day 15)	3	YrfF Y98D	Wzc D642G	MenB M56I	IS2 *ompT/pauD*	IS5 *htrL/rfaD*	
	4	YrfF Y98D	Wzc D642G	MenB M56I	IS2 *ompT/pauD*	IS5 *htrL/rfaD*	
	1	IS5 *csrA/alaS*	FrcK V211V				
Phenotype 3	2	IS5 *csrA/alaS*		*ynfF* +T(981/2424bp)			
(Day 30)	3	IS5 *csrA/ala*S					
	4	IS5 *csrA/alaS*					
	1	*mglC* (138bp)					
Phenotype 4	2	IS5 *mglC*					
(Day 30)	3	IS1 *mgl C*					
	4	BarA D472N					

Genes and amino acid sequence modifications are given for the SNPs. Mutations due to insertion sequences (IS) are described by the IS number and insertion position.

**Fig 3:**
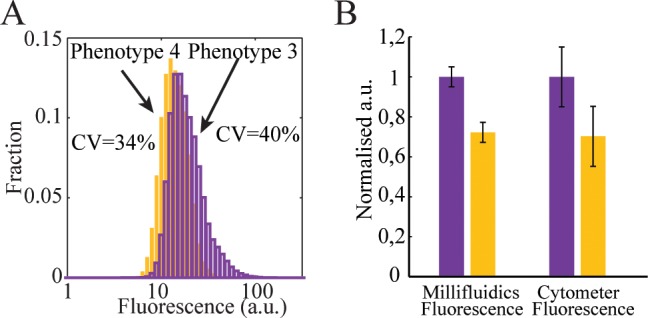
Comparison of YFP fluorescence obtained by cytometry and by lineage tracking. (A). Fluorescence distributions measured with a cytometer for phenotypes 3 and 4 isolated after 30 days of starvation (see Fig 2). Phenotype 4 (yellow bars) presents a mean single cell fluorescence of 13 arbitrary units (a.u.) with a CV of 34%. Phenotype 3 (purple bars) shows higher mean single cell fluorescence, 17 a.u., with a CV of 40%. The phenotypes are a posteriori discernible with a cytometer because they were isolated with the millifluidic tool prior to measurement. (B). Measurements of phenotypes 3 and 4 are characterized by: (i) millifluidics (left); (ii) flow cytometry (right). Phenotype 3 is purple, 4 is yellow. Error bars are standard deviations.

The individual drops corresponding to phenotypes 3 and 4 from the static microcosms (detected after 30 days of starvation), were also analysed by flow cytometry (Fig 3A) after recovery into wells of a microtitre plate. The difference in mean fluorescence between phenotypes 3 and 4 was almost identical when measured in the millifluidic system or using flow cytometry (Fig 3B), with phenotype 3 being 1.4-fold more fluorescent than phenotype 4. However, the CV of the fluorescent signal obtained using cytometry is large (Fig 3A), reflecting the impact of noise at the single cell level [17, 40, 41]. The high CV using flow cytometry makes discrimination of different phenotypic variants in the static microcosm (with mixed phenotypes) impossible, whereas they are clearly distinguishable using the millifluidic system. Indeed, direct analysis of the first static microcosm experiment by flow cytometry (S6 Fig) did not allow discrimination of distinct phenotypic classes, while lineage analysis using droplet millifuidics did reveal phenotypic clusters according to fluorescence time-curves (Fig 2), and or final fluorescence histograms (S6 Fig).

**Fig 4:**
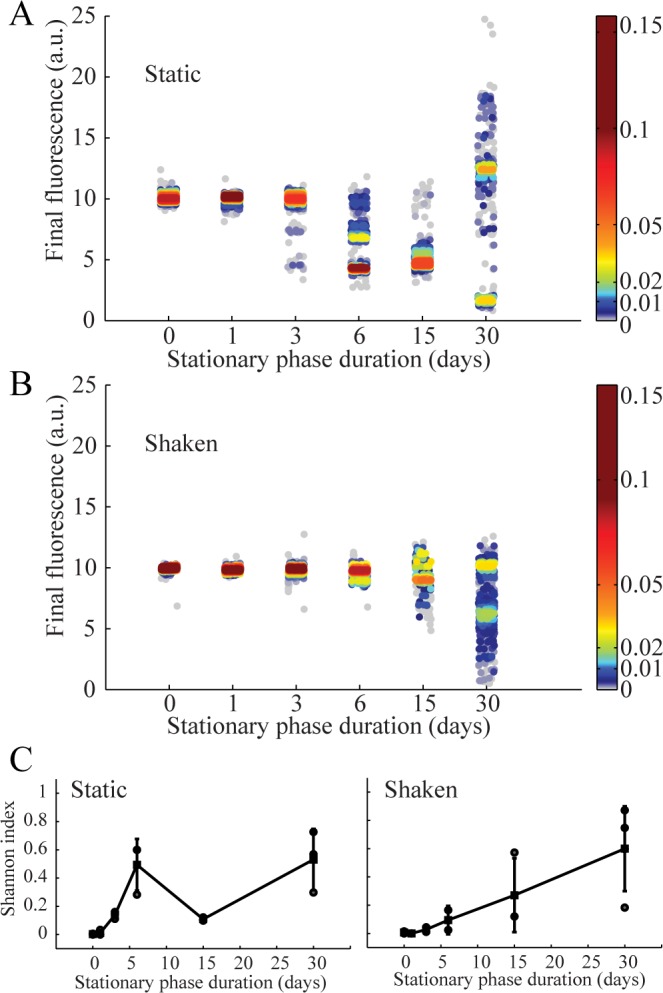
YFP fluorescence determined by lineage tracking and presented as a function of time spent in stationary phase for structured (static) microcosms (A, n = 3) and unstructured (shaken) microcosms (B, n = 3). (A) and (B) For a given duration (day X) the results for n = 3 replicates are merged within the same graph. Each dot corresponds to one observation and scattering around x-axis position corresponds to position within the droplet train. The y-axis position gives the measured final fluorescence levels normalized as described in the methods. The color scale corresponds to the observed frequency of the given fluorescence level at a given duration (~dots relative density). (C) Shannon index (as defined in the text), and as a function the duration of starvation: static (left) and shaken (right). Black squares correspond to the mean and gray points to each of the n = 3 experiments. Error bars correpond to CVs.

In Fig 4, the final yield fluorescence intensity levels measured in drops for a total of 30 independent starvation experiments are plotted as a function of time spent under static or shaken conditions. This provides a view of the diversification dynamics in the microcosms. For each sample, one to four discrete phenotypic classes are always present (Fig 2, S2 Fig). Moreover, the normalized fluorescence intensity and the frequency of the different phenotypic classes strongly fluctuate from one sample to another, even within a single time point (S2 Fig). Diversity is particularly large after 30 days, where fluorescence intensities vary more than 10 fold (Fig 4A). As a general feature, the number of phenotypic profiles (classes), their intensities, and the number of members in each class are found to strongly fluctuate from one experiment to another, confirming the existence of a range of possible evolutionary solutions and trajectories [2, 3, 21, 22]. Comparison with the results obtained for unstructured (shaken) microcosms (Fig 4B) confirms the importance of spatial structure for the process of diversification [9, 42]. When using the Shannon index [43] (see Methods) to quantify diversity between treatments (Fig 4C), early-stage diversification is higher under static relative to shaken conditions, but the final diversity is similar after 30 days in both conditions (Fig 4, S1 Fig). Thus, diversity under starvation builds up in about six days and then is dynamically maintained, as already suggested [21].

## Discussion

In contrast to approaches based on flow-cytometry [40, 41], time-lapse microscopy [17], or plating [44], our millifluidic system enables detection, quantitative analysis and isolation of clones. By tracking a signal reflecting the physiological state of the descendants of individual cells over about 15 generations, i.e. from a single cell to about 10^5^ cells, our methodology identifies heritable changes. Amplification across 15 generations overcomes gene expression noise occurring at each generation. According to the law of large numbers the contribution of this noise to the variance of our measurement scales as *σ*^2^/*N*, thus signal becomes more apparent with increasing generation number. In essence, the millifluidic system operates by a principle similar to used in agar plate culture as pioneered by Robert Koch in 1881 [45]. However, cells in colonies on plates experience different conditions depending on the proximity of colonies to one another, and depending upon the location of cells within colonies [44, 46, 47]. In contrast, in the millifluidic system, all individuals express their phenotype in strictly equivalent well-mixed environments. The phenotypic resolving-power of our method relies on the equivalence in growth and measurement conditions and capacity to amplify via growth, phenotypic differences that are indistinguishable at the single cell level.

Application of our methodology relied on the fluorescence signal derived from expression of *yfp* [17]. Expression of this gene is essentially constitutive except for the regulation by cAMP Receptor Protein (CRP). cAMP is a second messenger whose concentration depends on the growth medium, being in particular, sensitive to glucose levels (cAMP concentration is low when glucose is the carbon source). cAMP is also involved in regulation of numerous metabolic pathways [48] and therefore regulation of *yfp* by CRP stands as a reporter of the physiological state of the cell, and hence sensitive to many phenotypic changes. Thus our phenotypic trait of interest is the expression of *yfp* integrated over 15 generations. The detection of discrete classes in the starved static microcosm confirms the usefulness of the *yfp* reporter (Fig 2). The classes identified are strongly reminiscent of the consequences of evolution underpinned by genetic (mutational) change. Indeed, the emergence of clones with a fitness advantages associated with a heritable change would account for the occurrence of such classes. Although YFP fluorescence is not a direct measurement of fitness, phenotypic changes are nevertheless associated with measurable modifications of YFP fluorescence, allowing YFP fluorescence to be used to directly probe the dynamics of phenotypic diversity. By focussing on phenotypic variation across many generations of single cell lineages, our approach distinguishes variation in gene expression at the single cell level (noise) from heritable changes, such as those caused by mutations that are relevant for longer-term evolution. Evolutionary outcomes during long-term stationary phase have been shown to be sensitive to environmental conditions [49].

Here, we have demonstrated that a drop-based millifluidic system can be used to detect heritable phenotypic changes in evolving bacterial populations by tracking, in parallel, the lineage of descendants of single cells. By amplifying and averaging heritable changes over multiple generations variants, subtle phenotypic differences can be resolved in a way that is not possible using single-cell analysis techniques such as flow-cytometry. This approach offers a technological response to the growing need for reliable high-throughput phenotyping methods [50, 51] to keep pace with current theoretical developments and advances in genomics [52]. Furthermore, since individual drops can be sorted into microtitre plates, and analyzed by barcoded whole genome next-generation sequencing, it is feasible to map genotype to phenotype. While the proof of principle work described here was based on the analysis of ~500 single cells from each population, the technology is readily expanded to encompass the capture and analysis of the lineage of descendants of hundreds of thousands of cells.

The drop-based culturing strategy has utility in basic microbiology well beyond the simple application recounted here. It offers diverse applications in microbial physiology, ecology and evolution. For example, limiting dilutions of microbes from numerous sources can be readily partitioned among 1000s of drops (with the composition of drops being determined by the investigator), with growth from single cells being observed in real time, and independent populations then being available for genome sequencing, further phenotypic characterisation, and archiving. In addition, capacity to manipulate drops opens the door to a range of applications in experimental evolution and beyond.

## Materials and Methods

### Strains

*E*. *coli* MC41000-YFP [17], contains *yfp* at the *galK* locus under control of the *lac* promoter. The *lac* operon (including *lacI*) is deleted from the MC4100 background, thus expression is not regulated by the *lac* repressor and is essentially constitutive, but the *yfp* locus remains under CRP control.

### Culture conditions

All liquid cultures were in 5 mL of LB medium (Sigma-Aldrich) in closed polypropylene conical tubes (50 mL, VWR) and incubated at 37°C. First, 6 *E*. *coli* MC41000-YFP colonies were isolated on LB agar and then grown in liquid culture in closed tubes on an orbital shaker (VWR) at 100 rpm for five hours. Each of the 6 pre-cultures was used to inoculate 2x6 independent tubes (dilution 20x). Six tubes were incubated on an orbital shaker (VWR) at 100 rpm and six tubes were left static. Within each group of six tubes, the tubes were left in stationary phase for 1, 3, 6, 10, 15 and 30 days. At the end of the chosen duration each tube was mixed for homogeneous sampling. The samples were then stored at 80°C in 20% glycerol (VWR).

### Millifluidic droplet analyser

The analysis with the millifluidic lineage tracking machine was performed after dilution in fresh LB medium (Sigma-Aldrich) to obtain, an average number of cell per drop *λ* ≈ 0.5. Drops were incubated and measured at 37°C as described in [12, 13]. The fluorescence was measured using a photomultiplier (Hamamatsu) and a YFP filter set (479/40nm excitation and 530/40nm emission, Thorlabs) and an LED source (490nm, Thorlabs). In order to compare experiments, the data were normalized according to a reference measurement on the ancestral population.

### Viable cell density determination

Viable cell density measured at day 1, 3, 6, 10, 15 and 30 was estimated using the millifluidic analyser. When generating drops, the cell encapsulation probability follows the Poisson law. If the average number of viable cells per drop is *λ*, the probability to form a drop with no bacteria is exp(−*λ*). This probability is estimated according to the fraction of drops with no detectable growth, *x*_empty_: λ∼− ln(*x*_empty_). Knowing the drop volume, we can finally determine the viable cells density within the sample: *cfu* = λ.V_*drop*_.

### Colony morphology and Cytometry

Isolated clones (S3 Fig) of new phenotypes were plated on LB agar (BD). Colony morphologies were compared after 15h of incubation at 37°C. In order to test the relationship between fluorescence and the biomass, different isolated phenotypes were analysed by cytometry. Comparison of measurements as shown in Fig 3 were obtained for populations of each phenotype grown separately under the same conditions: 37°C shaking at 100 rpm (cultures were grown to stationary phase). Samples were then analyzed separately after dilution in phosphate buffered saline using a Partec cytometer (CyFlow® Space).

### Genome sequencing

The ancestral and 16 isolated clones (4 clones per phenotype) were grown overnight in 5 ml of M9. DNA was purified from these cultures using a bacterial genomic DNA kit (Sigma-Aldrich). DNA library construction and sequencing were carried out at the NGS platform of the Institut Curie. The 16 clones were DNA barcoded and paired-end sequenced (2 x 150 bp reads) in a single run on an Illumina HiSeq 2500. Mutations in the genomes were identified using the computational pipeline BRESEQ [53]. Accession number on SRA database at NCBI is SRX1564688.

### Shannon index

The Shannon index [43] is defined as ∑ *p*_*i*_.*ln*(*p*_*i*_) where p are the observed occurrences of phenotypic classes, i.e. pi=niN, where *n*_*i*_ is the observed number of the i-th phenotype and N the total number of observed cells.

## Supporting Information

S1 Fig YFP fluorescence profiles obtained by lineage tracking (shaken microcosms). Samples of ~200 cells from independent evolving populations from a single replicated experiment were obtained for the ancestor population and after 1, 3, 6, 15 and 30 days of starvation in shaken microcosms (from top to bottom), and grown for ∼13 h in individual droplets. A.U. is arbitrary units. Measurement of YFP fluorescence from each droplet was measured every 10 min. Colors discriminate phenotypic classes.
(TIF)

S2 Fig Millifluidic single-cell lineage tracking obtained for 18 independent samples. Six independent initial colonies (colony 1–6); distinct samples were analysed after 6, 15 or 30 days of stationary phase (column). Graphs for colony 1 are also shown in Fig 2 in the main text. Colors discriminate phenotypic classes.
(TIF)

S3 Fig Comparison of YFP Fluorescence obtained by cytometry and by lineage tracking for the ancestral population. (A). Single cell fluorescence distribution for the ancestor population measured by flow cytometry. Data are plotted on a logarithmic scale, the coefficient of variation (CV) is 34%. (B). Distribution of the final fluorescence of profiles obtained for the ancestor population by lineage tracking across ∼500 drops. Data are reported on a linear scale, the CV is 5%.
(TIF)

S4 Fig Schematic and photograph of the sorting module. Arrows link corresponding parts within the two representations. Drops to be sorted are identified by their position within the 1D sequence. In order to dispense the chosen drops into the microplate wells, the distance between drops is increased (by injecting additional fluorocarbon oil with T1, T2 and T3 “T”-junctions); then the entire train is directed toward the open end of a PTFE tube. The open end is kept above a waste container until the signal of selected drop is transmitted (labelled by position). At that time the flow is stopped until the XY automated stage positions the tube termination above a defined well, then the drop is collected.
(TIF)

S5 Fig The phenotypic classes observed after 6, 15 and 30 days show different colony morphologies on LB-agar. The ancestor produces flat colonies (top left), phenotype 1 and 2 produces mucoid colonies (top right), the photograph was obtained with low transmission light to reveal the reflection on mucoid colonies). Phenotypes 3 and 4 isolated after 30 days of starvation produce colonies of different sizes. Phenotypes 3 and 4 both produce non-mucoid colonies. Phenotype 4 (bottom right), which reaches a lower final fluorescence signal (see Fig 2), produces larger colonies. Phenotype 3 (bottom left) produces smaller colonies in agreement with the slower apparent growth rate observed with the fluorescence measured in drops (see Fig 2). Scale bars = 5mm.
(TIF)

S6 Fig Histograms of the final YFP Fluorescence measured in the Millifluidic Droplet Analyser and YFP Fluorescence obtained by flow cytometry for populations after prolonged starvation. Final fluorescence signal histograms obtained with the Millifluidic Droplet Analyser (left) and Fluorescence distributions obtained by flow cytometry (right). These histograms are obtained on the same samples as on Fig 2 obtained after 1, 3, 6, 15 and 30 days of starvation (from top to bottom). The coefficient of variation for the distributions on the right varies between 0.45 and 0.49.
(TIF)

S1 Movie Still image of S1 Movie showing drop generations process. (AVI)

S2 Movie Still image of S2 Movie showing drops moving back and forth at the fluorescence detection position. (AVI)

## Abbreviations

GASP: growth advantage in stationary phase
CV: coefficient of variation
YFP: Yellow Fluorescent Protein
OD: Optical Density

## References

[1] Elena SF, Lenski RE. Microbial genetics: Evolution experiments with microorganisms: the dynamics and genetic bases of adaptation. Nat. Rev. Genet. 4(6), 457–469 (2003). 12776215 10.1038/nrg1088

[2] Kawecki TJ, Lenski RE, Ebert D, Hollis B, Olivieri I, Whitlock MC. Experimental evolution. Trends Ecol. Evol. 27(10), 547–560 (2012). 10.1016/j.tree.2012.06.001 22819306

[3] Lenski RE, Rose MR, Simpson SC, Tadler SC. Long-term experimental evolution in Escherichia coli. I. Adaptation and divergence during 2,000 generations. Am. Nat. 138(6), 1315–1341 (1991).

[4] Barrick JE, Lenski RE. Genome dynamics during experimental evolution. Nat. Rev. Genet. 14(12), 827–839 (2013). 10.1038/nrg3564 24166031 PMC4239992

[5] Levy SF, Blundell JR, Venkataram S, Petrov DA, Fisher DS, Sherlock G. Quantitative evolutionary dynamics using high-resolution lineage tracking. Nature 519(7542), 181–186 (2015). 10.1038/nature14279 25731169 PMC4426284

[6] Beaumont HJE, Gallie J, Kost C, Ferguson GC, Rainey PB. Experimental evolution of bet hedging. Nature 462(7269), 90–93 (2009). 10.1038/nature08504 19890329

[7] Gallie J, Libby E, Bertels F, Remigi P, Jendresen CB, Ferguson GC, et al Bistability in a metabolic network underpins the de novo evolution of colony switching in Pseudomonas fluorescens. PLoS Biol. 13(3), e1002109 (2015). 10.1371/journal.pbio.1002109 25763575 PMC4357382

[8] Lind PA, Farr AD. Rainey PB. Experimental evolution reveals hidden diversity in evolutionary pathways. eLife 4, e07074 (2015). 10.7554/eLife.07074 PMC4395868 25806684

[9] Rainey PB, Travisano M. Adaptive radiation in a heterogeneous environment. Nature 394(6688), 69–72 (1998). 9665128 10.1038/27900

[10] Plucain J, Hindré T, Le Gac M, Tenaillon O, Cruveiller S, Médigue C, et al Epistasis and allele specificity in the emergence of a stable polymorphism in Escherichia coli. Science 343(6177), 1366–1369 (2014). 10.1126/science.1248688 24603152

[11] Hansen SK, Rainey PB, Haagensen JAJ, Molin S. Evolution of species interactions in a biofilm community. Nature 445(7127), 533–536 (2007). 17268468 10.1038/nature05514

[12] Baraban L, Bertholle F, Salverda ML, Bremond N, Panizza P, Baudry J, et al Millifluidic droplet analyser for microbiology. Lab Chip 11(23), 4057–4062 (2011). 10.1039/c1lc20545e 22012599

[13] Damodaran SP, Eberhard S, Boitard L, Rodriguez JG, Wang Y, Bremond N, et al A Milliffuidic Study of Cell-to-Cell Heterogeneity in Growth-Rate and Cell-Division Capability in Populations of lsogenic Cells of Chlamydonnnas reinhardtii. PLoS ONE 10(3): e0118987 (2015) 10.1371/journal.pone.0118987 25760649 PMC4356620

[14] Jakiela S, Kaminski TS, Cybulski O, Weibel DB, Garstecki P. Bacterial growth and adaptation in microdroplet chemostats. Angew. Chem. Int. Ed., 52: 8908–8911., (2013). 10.1002/anie.201301524 PMC3879160 23832572

[15] Boedicker JQ, Vincent ME, Ismagilov RF. Microfluidic Confinement of Single Cells of Bacteria in Small Volumes Initiates High-Density Behavior of Quorum Sensing and Growth and Reveals Its Variability. Angew. Chem. Int. Ed., 48: 5908–5911 (2009) 10.1002/anie.200901550 PMC2748941 19565587

[16] Bachmann H, Fischlechner M, Rabbers I, Barfa N, Branco dos Santos F, Molenaar D, et al Availability of public goods shapes the evolution of competing metabolic strategies, PNAS, 110 (35), 14302–14307, (2013) 10.1073/pnas.1308523110 23940318 PMC3761572

[17] Elowitz MB, Levine AJ, Siggia ED, Swain PS. Stochastic Gene Expression in a Single Cell. Science 297(5584), 1183–1186 (2002). 12183631 10.1126/science.1070919

[18] Hegreness M, Shoresh N, Hartl D, Kishony R. An equivalence principle for the incorporation of favorable mutations in asexual populations. Science 311(5767), 1615–1617 (2006). 16543462 10.1126/science.1122469

[19] Naas T, Blot M, Fitch WM, Arber W. Insertion sequence-related genetic variation in resting Escherichia coli K-12. Genetics 136(3), 721–730 (1994). 7911771 10.1093/genetics/136.3.721 PMC1205879

[20] Zambrano M, Siegele D, Almiron M, Tormo A, Kolter R. Microbial competition: Escherichia coli mutants that take over stationary phase cultures. Science 259(5102), 1757–1760 (1993). 7681219 10.1126/science.7681219

[21] Finkel SE. Long-term survival during stationary phase: evolution and the GASP phenotype. Nat. Rev. Microbiol. 4(2), 113–120 (2006). 16415927 10.1038/nrmicro1340

[22] De Visser JAGM, Rozen DE. Clonal interference and the periodic selection of new beneficial mutations in Escherichia coli. Genetics 172(4), 2093–2100 (2006). 16489229 10.1534/genetics.105.052373 PMC1456385

[23] Poltak SR, Cooper VS. Ecological succession in long-term experimentally evolved biofilms produces synergistic communities. ISME J. 5(3), 369–378 (2011). 10.1038/ismej.2010.136 20811470 PMC3105725

[24] Callahan BJ, Fukami T, Fisher DS. Rapid evolution of adaptive niche construction in experimental microbial populations. Evolution 68(11), 3307–3316 (2014). 10.1111/evo.12512 25138718

[25] Choi K, Ng AHC, Fobel R, Wheeler AR. Digital microfluidics. Annu. Rev. Anal. Chem. 5(1), 413–440 (2012). 10.1146/annurev-anchem-062011-143028 22524226

[26] Theberge AB, Courtois F, Schaerli Y, Fischlechner M, Abell C, Hollfelder F, et al Microdroplets in microfluidics: an evolving platform for discoveries in chemistry and biology. Angew. Chem. Int. Ed. 49, 5846–5868 (2010). 10.1002/anie.200906653 20572214

[27] Köster S, Angilè FE, Duan H, Agresti JJ, Wintner A, Schmitz C, et al Drop-based microfluidic devices for encapsulation of single cells. Lab Chip 8(7), 1110–1115 (2008). 10.1039/b802941e 18584086

[28] Clausell-Tormos J, Lieber D, Baret JC, El-Harrak A, Miller OJ, Frenz L, et al Droplet-based microfluidic platforms for the encapsulation and screening of Mammalian cells and multicellular organisms. Chem. Biol., 15(5), 427–437 (2008). 10.1016/j.chembiol.2008.04.004 18482695

[29] Klumpp S, Zhang Z, Hwa T. Growth-rate dependent global effects on gene expression in bacteria. Cell 139(7), 1366 (2009). 10.1016/j.cell.2009.12.001 20064380 PMC2818994

[30] Beiser SM, Davis BD. Mucoid mutants of Escherichia coli. J. Bacteriol. 74(3), 303–307 (1957). 13475241 10.1128/jb.74.3.303-307.1957 PMC314639

[31] Miskinyte M, Sousa A, Ramiro RS, de Sousa JA, Kotlinowski J, Caramalho I, et al The genetic basis of Escherichia coli pathoadaptation to macrophages. PLoS Pathog. 9(12), e1003802 (2013). 10.1371/journal.ppat.1003802 24348252 PMC3861542

[32] Wichman HA, Badgett MR, Scott LA, Boulianne CM, Bull JJ. Different trajectories of parallel evolution during viral adaptation. Science 285(5426), 422–424 (1999). 10411508 10.1126/science.285.5426.422

[33] Obadia B, Lacour S, Doublet P, Baubichon-Cortay H, Cozzone AJ, Grangeasse C. Influence of tyrosine-kinase Wzc activity on colanic acid production in Escherichia coli K12 cells. J. Mol. Biol. 367(1), 42–53 (2007). 17254603 10.1016/j.jmb.2006.12.048

[34] Zinser ER, Schneider D, Blot M, Kolter R. Bacterial evolution through the selective loss of beneficial genes: trade-offs in expression involving two loci. Genetics 164(4), 1271–1277 (2003). 12930738 10.1093/genetics/164.4.1271 PMC1462639

[35] Zinser ER, Kolter R. Prolonged stationary-phase incubation selects for lrp mutations in Escherichia coli K-12. J. Bacteriol. 182(15), 4361–4365 (2000). 10894750 10.1128/jb.182.15.4361-4365.2000 PMC101964

[36] Mukhopadhyay S, Audia JP, Roy RN, Schellhorn HE. Transcriptional induction of the conserved alternative sigma factor RpoS in Escherichia coli is dependent on BarA, a probable two-component regulator. Mol. Microbiol. 37, 371–338 (2000). 10931332 10.1046/j.1365-2958.2000.01999.x

[37] Suzuki K, Wang X, Weilbacher T, Pernestig AK, Melefors O, Georgellis D, et al Regulatory circuitry of the CsrA/CsrB and BarA/UvrY systems of Escherichia coli. J. Bacteriol. 184(18), 5130–5140 (2002). 12193630 10.1128/JB.184.18.5130-5140.2002 PMC135316

[38] Camacho MI, Alvarez AF, Chavez RG, Romeo T, Merino E, Georgellis D. Effects of the global regulator CsrA on the BarA/UvrY two-component signaling system. J. Bacteriol 197(5), 983–991 (2015). 10.1128/JB.02325-14 25535275 PMC4325108

[39] Romeo T. Global regulation by the small RNA-binding protein CsrA and the non-coding RNA molecule CsrB. Molecular Microbiology 29(6), 1321–1330 (1998). 9781871 10.1046/j.1365-2958.1998.01021.x

[40] Ozbudak EM, Thattai M, Kurtser I, Grossman AD, van Oudenaarden A. Regulation of noise in the expression of a single gene. Nat. Genet. 31(1), 69–73 (2002). 11967532 10.1038/ng869

[41] Brenner N, Farkash K, Braun E. Dynamics of protein distributions in cell populations. Phys. Biol. 3(3), 172 (2006). 17021381 10.1088/1478-3975/3/3/002

[42] Habets MGJL, Rozen DE, Hoekstra RF, De Visser JAGM. The effect of population structure on the adaptive radiation of microbial populations evolving in spatially structured environments: Spatial structure and adaptive radiation. Ecol. Lett. 9(9), 1041–1048 (2006). 16925653 10.1111/j.1461-0248.2006.00955.x

[43] Hill MO. Diversity and evenness: a unifying notation and its consequences. Ecology 54(2), 427–432. (1973)

[44] Levin-Reisman I, Gefen O, Fridman O, Ronin I, Shwa D, Sheftel H, et al Automated imaging with ScanLag reveals previously undetectable bacterial growth phenotypes. Nat. Meth. 7(9), 737–739 (2010). 10.1038/nmeth.1485 20676109

[45] Koch R. Zur Untersuchung von pathogenen Organismen. Mittheilungen aus dem Kaiserlichen Gesundbeitsamte, vol. 1, 1–48 (1881).

[46] Pirt SJ. A kinetic study of the mode of growth of surface colonies of bacteria and fungi. J. Gen. Microbiol. 47(2), 181–197 (1967). 6045659 10.1099/00221287-47-2-181

[47] Wimpenny JWT. The growth and form of bacterial colonies. J. Gen. Microbiol. 114(2), 483–486 (1979). 120410 10.1099/00221287-114-2-483

[48] Shimada T, Fujita N, Yamamoto K, Ishihama A. Novel roles of cAMP Receptor Protein (CRP) in regulation of transport and metabolism of carbon sources. PLoS ONE 6(6), e20081 (2011) 10.1371/journal.pone.0020081 21673794 PMC3105977

[49] Kram KE, Finkel SE. Culture volume and vessel affect long-term survival, mutation frequency, and oxidative stress of Escherichia coli. Appl. Environ. Microbiol. 80(5), 1732–1738 (2014). 10.1128/AEM.03150-13 24375138 PMC3957596

[50] Desai MM. Statistical questions in experimental evolution. J. Stat. Mech. 2013(01), P01003 (2013)

[51] Lang GI, Rice DP, Hickman MJ, Sodergren E, Weinstock GM, Botstein D, et al Pervasive genetic hitchhiking and clonal interference in forty evolving yeast populations. Nature 500, 571–574 (2010) 10.1038/nature12344 PMC3758440 23873039

[52] Prosser JI, Bohannan B, Curtis TP, Ellis RJ, Firestone MK, Freckleton RP, et al The role of ecological theory in microbial ecology. Nat. Rev. Microbiol. 5(5), 384–392 (2007) 17435792 10.1038/nrmicro1643

[53] Barrick J, Yu DS, Yoon SH, Jeong H, Oh TK, Schneider D, et al Genome evolution and adaptation in a long-term experiment with Escherichia coli. Nature 461 (7268), 1243–1247 (2009) 10.1038/nature08480 19838166

