# Supplementary figures and images for: Lineage Tracking for Probing Heritable Phenotypes at Single-Cell Resolution
Source: PLoS One. 2016 Apr 14;11(4):e0152395. doi: 10.1371/journal.pone.0152395 (PMC4831777; doi:10.1371/journal.pone.0152395)

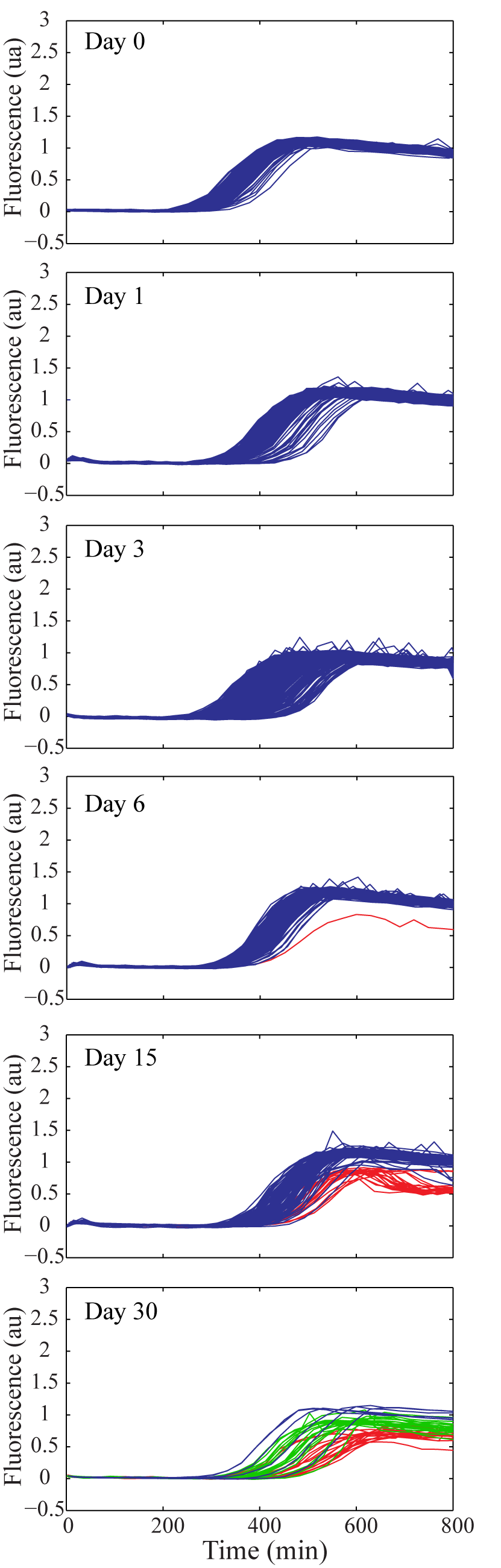

Supplement: S1 Fig — Samples of ~200 cells from independent evolving populations from a single replicated experiment were obtained for the ancestor population and after 1, 3, 6, 15 and 30 days of starvation in shaken microcosms (from top to bottom), and grown for ∼13 h in individual droplets. A.U. is arbitrary units. Measurement of YFP fluorescence from each droplet was measured every 10 min. Colors discriminate phenotypic classes. (TIF) [file pone.0152395.s001.tif]

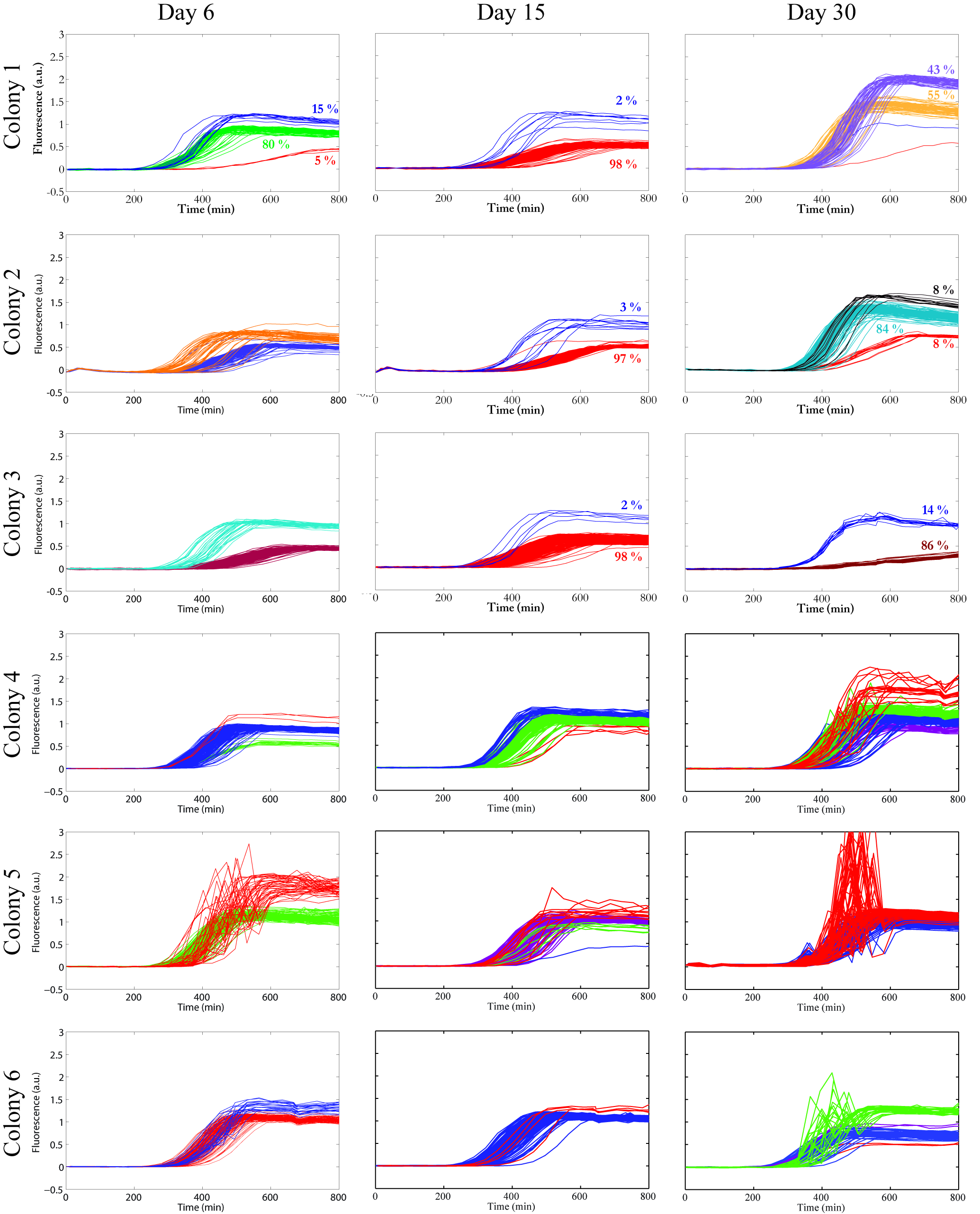

Supplement: S2 Fig — Six independent initial colonies (colony 1–6); distinct samples were analysed after 6, 15 or 30 days of stationary phase (column). Graphs for colony 1 are also shown in Fig 2 in the main text. Colors discriminate phenotypic classes. (TIF) [file pone.0152395.s002.tif]

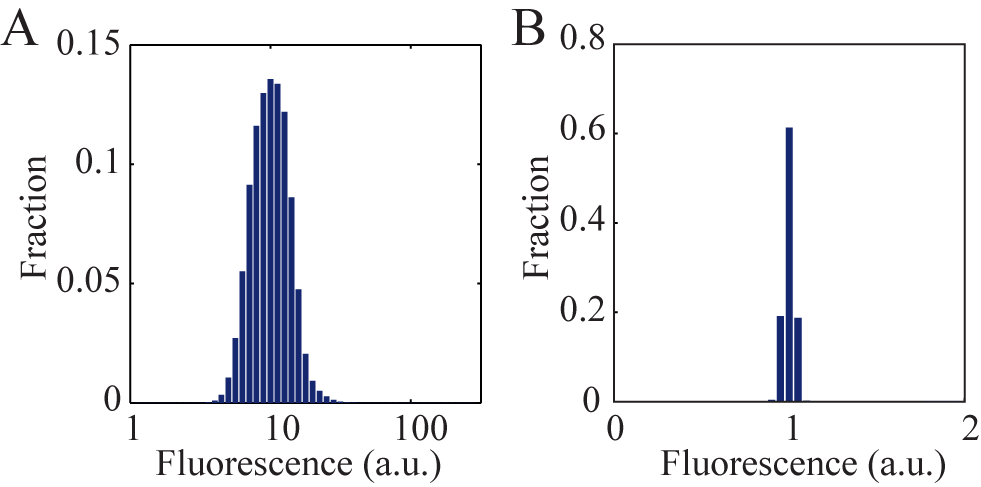

Supplement: S3 Fig — (A). Single cell fluorescence distribution for the ancestor population measured by flow cytometry. Data are plotted on a logarithmic scale, the coefficient of variation (CV) is 34%. (B). Distribution of the final fluorescence of profiles obtained for the ancestor population by lineage tracking across ∼500 drops. Data are reported on a linear scale, the CV is 5%. (TIF) [file pone.0152395.s003.tif]

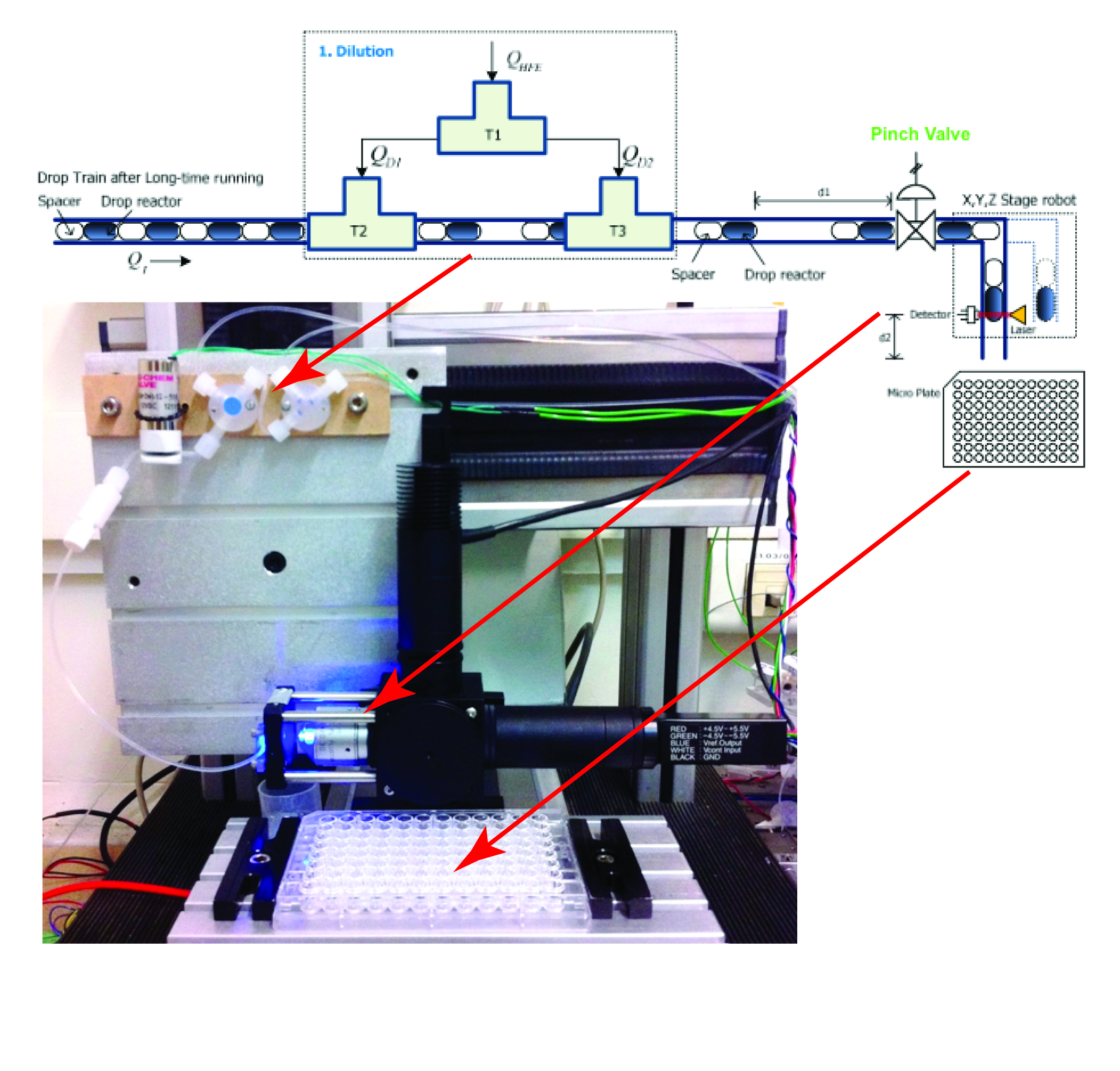

Supplement: S4 Fig — Arrows link corresponding parts within the two representations. Drops to be sorted are identified by their position within the 1D sequence. In order to dispense the chosen drops into the microplate wells, the distance between drops is increased (by injecting additional fluorocarbon oil with T1, T2 and T3 “T”-junctions); then the entire train is directed toward the open end of a PTFE tube. The open end is kept above a waste container until the signal of selected drop is transmitted (labelled by position). At that time the flow is stopped until the XY automated stage positions the tube termination above a defined well, then the drop is collected. (TIF) [file pone.0152395.s004.tif]

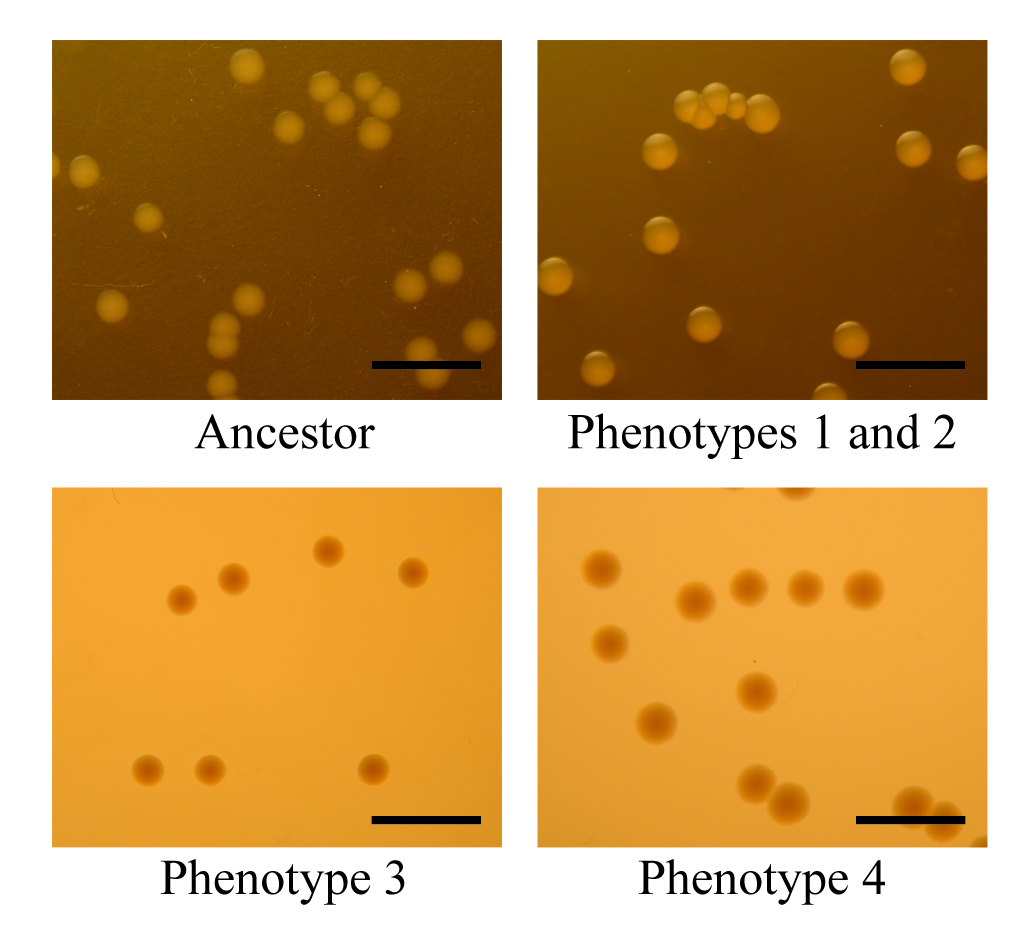

Supplement: S5 Fig — The ancestor produces flat colonies (top left), phenotype 1 and 2 produces mucoid colonies (top right), the photograph was obtained with low transmission light to reveal the reflection on mucoid colonies). Phenotypes 3 and 4 isolated after 30 days of starvation produce colonies of different sizes. Phenotypes 3 and 4 both produce non-mucoid colonies. Phenotype 4 (bottom right), which reaches a lower final fluorescence signal (see Fig 2), produces larger colonies. Phenotype 3 (bottom left) produces smaller colonies in agreement with the slower apparent growth rate observed with the fluorescence measured in drops (see Fig 2). Scale bars = 5mm. (TIF) [file pone.0152395.s005.tif]

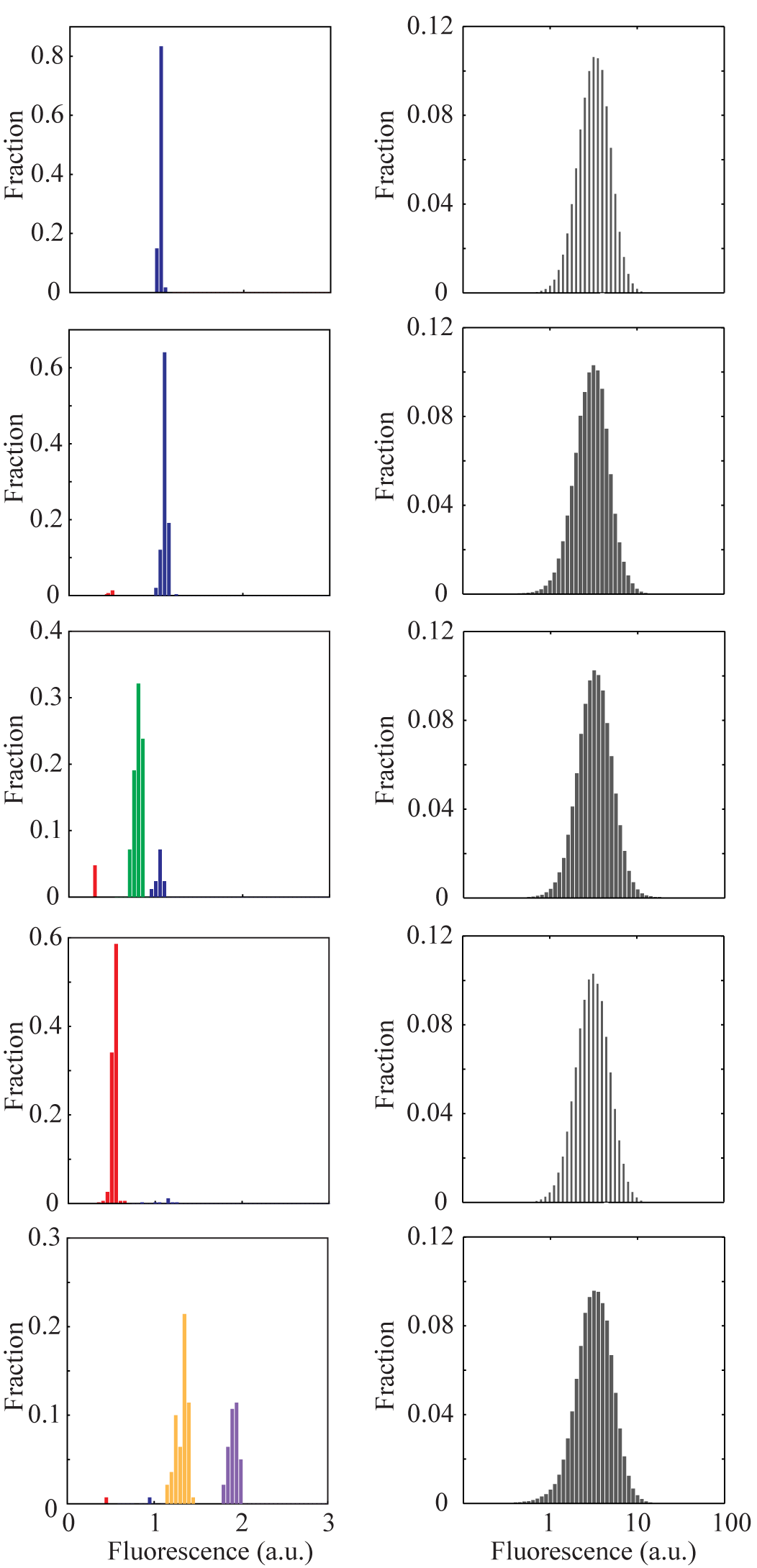

Supplement: S6 Fig — Final fluorescence signal histograms obtained with the Millifluidic Droplet Analyser (left) and Fluorescence distributions obtained by flow cytometry (right). These histograms are obtained on the same samples as on Fig 2 obtained after 1, 3, 6, 15 and 30 days of starvation (from top to bottom). The coefficient of variation for the distributions on the right varies between 0.45 and 0.49. (TIF) [file pone.0152395.s006.tif]
